# Reasons for non- use of condoms and self- efficacy among female sex workers: a qualitative study in Nepal

**DOI:** 10.1186/1472-6874-11-42

**Published:** 2011-09-26

**Authors:** Laxmi Ghimire, W Cairns S Smith, Edwin R van Teijlingen, Rashmi Dahal, Nagendra P Luitel

**Affiliations:** 1 School of Medicine, Public Health Department, University of Aberdeen, Scotland, UK; 2 School of Health and Social Care, Bournemouth University, Royal London House, Bournemouth, BH1 3LT, UK; 3 Institute of Medicine, Maharajgunj campus, Tribhuvan University, Kathmandu, Nepal; 4 Transcultural Psychosocial Organisation (TPO), Baluwatar Kathmandu, Nepal

## Abstract

**Background:**

Heterosexual contact is the most common mode of transmission of sexually transmitted infections (STIs) including Human Immunodeficiency Virus (HIV) in Nepal and it is largely linked to sex work. We assessed the non-use of condoms in sex work with intimate sex partners by female sex workers (FSWs) and the associated self-efficacy to inform the planning of STI/HIV prevention programmes in the general population.

**Methods:**

This paper is based on a qualitative study of Female Sex Workers (FSWs) in Nepal. In-depth interviews and extended field observation were conducted with 15 FSWs in order to explore issues of safe sex and risk management in relation to their work place, health and individual behaviours.

**Results:**

The main risk factor identified for the non-use of condoms with intimate partners and regular clients was low self efficacy. Non-use of condoms with husband and boyfriends placed them at risk of STIs including HIV. In addition to intimidation and violence from the police, clients and intimate partners, clients' resistance and lack of negotiation capacity were identified as barriers in using condoms by the FSWs.

**Conclusion:**

This study sheds light on the live and work of FSWs in Nepal. This information is relevant for both the Government of Nepal and Non Governmental Organisations (NGO) to help improve the position of FSWs in the community, their general well-being and to reduce their risks at work.

## Background

Sex work is characterised by high rates of commercial sex partner exchange, low rates of consistent condom use [1] with regular partners (only 5.9%) and with last sex client (66.3%) [2]. The conditions and environment of sex work in South East Asia have not been well described, despite rising sexually transmitted infections (STI) and human immunodeficiency virus (HIV) incidence rates which are attributed to both sex work and drug use [3,4].

Nepal is one of the least developed country, many people live in poverty and the country experiences considerable seasonal labour migration [5] mainly to India and the Middle East. Labour migrants to India constitute one of the 'bridging populations' for the transmission of STIs and HIV infection. One specific element of this labour migration is that Nepalese women end up working as Female Sex Workers (FSWs) in the big cities in India. It is estimated that some 200,000 Nepalese women work in the sex industry in India.

Moreover, the conditions in which FSWs operate need to be seen in the light of the inferior position of women in Nepalese society [6]. Not only is Nepal a very patriarchal society, it is also a society where talking about sex is still a taboo [7]. As in many countries across the world sex work or prostitution is an illegal activity in Nepal.

Nepal generally has a low HIV prevalence of less than one percent, but it is considerably higher in FSWs, whose prevalence rate is 4% nationally [8]. In and around the capital in Kathmandu Valley, the HIV prevalence in street based FSWs is reported to be 17% [9,10] and the HIV rate among Nepalese sex-trafficked girls and women is 38.0%, which is very high within South Asia [11].

The higher client-FSW ratio (20:1) in Nepal, compared to the South Asian average (7.5:1) indicates higher risks of STIs and HIV transmission among the FSWs including the general population [11]. The situation is likely to be aggravated due to FSWs' low socio-economic and education status, stigma and discrimination, and the high level of violence from intimate partners associated with their sex work [12-15]. Violence and stigma are even higher than that experienced by women generally in Nepal [16,17] and coupled with the fact that sex work is illegal; create barriers to the consistent use of condoms.

In Nepal, one study concluded that clients did not want to wear condoms for fear of losing sexual pleasure and embarrassment over buying condoms [18]. FSW are less likely to seek diagnosis and treatment for symptoms of STIs by a medical professional due to fear of exposure by health workers or other authority figures [19-21].

This paper presents individual, structural and cultural factors facilitating and creating barriers in using condoms among FSWs in the Kathmandu Valley.

## Methods

### Study sample and methods

Our qualitative study focused on FSWs aged 15 to 45 who have been involved in the commercial sex trade for at least six months prior to the research. FSWs constitute a hard-to- reach population [22,23]. We conducted 15 in-depth interviews from a purposely selected sub-sample from 425 FSWs who had participated in a questionnaire survey on sex work [21]. These 425 FSWs were interviewed individually using a quantitative, structured questionnaire, and 15 FSWs participated in a second, qualitative, in-depth interview out of the 25 FSWs who were invited to participate. This second set of interviews aimed to complement the information collected from the short individual interviews, exploring in depth areas such as using condoms with paying clients and non-paying partners and risk taking behaviour, which were not accurately answered in the quantitative survey. Efforts were made to select a diversity of respondents in terms of age, marital status, ethnicity, and socioeconomic status to obtain a range of opinions and experiences.

An interview schedule was prepared outlining some topics to be discussed and some probing questions. This was first developed in English and translated into Nepali. Interview topics included knowledge and use of condoms, sexual activities and protective behaviour, potential partners, sexual harassment, characteristic of paying clients and non-paying partners, and high-risk sexual behaviour. The interviews were tape recorded with written consent. Three participants refused to participate as information about them had been exposed by the media in the past. Theoretical saturation was reached after 15 interviews and no new additional information, appeared after the 13^th ^interview [24]. Interviewing is appropriate for addressing sensitive issues [25]. Conducting interviews allows the researcher some control over the line of questioning, whilst still leaving scope for respondents to cover issues not addressed in the interview schedule.

The first author conducted the in-depth interviews, with the help from two trained researchers, at convenient locations and at dates and times of the interviewee's choice, usually in a public area due to fear of disclosure of their occupation. The in-depth interviews completed by the first author lasted between two to five hours and several visits were made prior to the interview to build up rapport with the interviewee.

### Ethical considerations

Ethical approval was given by the Nepal Health Research Council (NHRC). Participants' full verbal and written informed consent was obtained; using consent forms written in Nepali and using simple terminology. Those respondents who requested additional information were referred to the nearest health institutions. Confidentiality of information was maintained by removing personal identifiers, names of hotels and massage centres where FSWs operate. The interviews were analysed manually through reading and re-reading of the transcripts and manual thematic analysis by two of the authors.

## Results

### FSW Socio-demographic Characteristics

Of the 15 FSWs interviewed six were married and living with their husbands, five were married but separated, three were unmarried, and one was a widow. Of those who were married, their marriages were well established (15 to 17 years). The majority were Janajati (ethnic minority group), two were Dalits (disadvantaged low castes), two were Brahman/Chhetri (privileged castes), and one was a Newar (privileged ethnic group). Their ages ranged from 19 to 42 years, with most (80%) being between 22 and 30 years old. In order to keep the identity of the FSWs confidential, each was assigned identification (ID). The interviewees' characteristics are listed in Table 1 by number and these numbers are used in the quotes below as identifiers.

**Table 1:** Demographic Characteristics of the FSWs Interviewed

ID No.	Age	Marital status	Education (highest level)	Place of work/where based
1	28	Married	Grade 5	NGO outreach worker
2	19	Separated	Grade 9	Restaurant dancer
3	24	Married	Illiterate	Street
4	39	single	Illiterate	Restaurant worker
5	22	Separated	Illiterate	Cabin Restaurant
6	22	Married	Literate	Street
7	42	Widowed	Literate	Massage parlour owner centre
8	30	Married	Literate	Massage parlour owner centre
9	25	Married	Literate	Street
10	26	Single	Grade 9	Local bar/restaurant
11	30	Separate	Grade 5	Restaurant worker
12	24	Single	Grade 10	Hotel call girl
13	22	Separate	Literate	Restaurant worker
14	30	Married	Literate	NGO outreach worker educator
15	30	Single	Literate	Street

### Self-efficacy (SE) and Condoms Use

All FSWs knew about condoms. They also claimed that they knew how to use condoms during sexual intercourse which meant a high self-efficacy (SE) with FSWs in this matter. Two peer educators and the owner of a massage centre were involved in educating other FSWs on how to use condoms. It was notable that almost all FSWs reported they had not used condoms. Eleven out of 15 said they did not use condoms with their most recent client because of the client's refusal. Four out of 15 self reported that they had used condoms in their last sexual encounter, but when they were probed further, they revealed that they actually had not used condoms with their last client. The reasons for non use of condoms were reported as clients refused in low SE.

### Client refusal

Almost all FSWs spoke of client refusing to use condoms for reasons of reduced pleasure, or that they knew each other well and had an established relationship. In fact, FSWs used condoms only if their clients demanded that they use them, the clients generally did not demand that condoms to be used. In cases of client refusal, FSWs did not disagree or try to force clients because they feared that they would lose the client if they disagreed to have sex. The following quote is typical in that it highlights the client's payment for pleasure and the FSW's in ability to refuse on the grounds of unsafe sex:

*'I asked him (client) to use a condom. He replied that he did not like them and wanted more pleasure while having sex. He further added that he had come to relax as he had paid the restaurant owner already, I could not deny him sex anyway' (*FSW ID 5, age 22)

Another FSW stated clearly that condom use depended on the client's attitude, for example:

*'I ask every client to use a condom. If he accepts I use the condom. If he does not, I cannot force him. Condom use really depends on the client' *(FSW ID 11, age 30)

Two FSWs felt that they were more educated and fashionable than other FSWs, and had clients who were of higher status: therefore their exposure to STIs and HIV was reduced.

*'I use condoms with foreigners as they demand them. But with other high profile Nepali men, I do not use them' *(FSW ID 7, age 42)

Consistent condom use was claimed by just one FSW with a higher education and better employment in a dance restaurant:

*'I am an attractive lady. I have passed my school leaving certificate (Grade 10). I work in a dance restaurant and I know many good looking and high class men. I charge more than Rs.2000 per client. I use condoms every time. If a client refuses to use a condom, I get another client' *(FSW ID 12, age 24)

Almost all FSWs reported that they never asked their boyfriends and husband to use condoms except for family planning purpose. They do not use condoms consistently with their regular sex partners, or the husband, Nepali FSWs expressed their powerlessness to force their husband to use condoms against their will. Even a FSW who was (a) a peer educator; (b) and living with an HIV positive husband did not use condoms with either clients or her husband.

She said that she knew the risk but could not stop people having sex without condoms.

*'I know the danger of having sex without condoms, my husband is HIV positive, and how can I say this to other clients? How can I stop my husband having sex without a condom? I am helpless' *(FSW ID 1, age 28)

### Decision-making in condom use

We asked FSWs who decided whether or not to use a condom in their last sexual encounter. Of the 15 FSWs interviewed, 6 FSWs reported that it was decided by them, 3 by the client, and the remainder by both of them. One FSW interviewee noted that she never use condom and neither she nor her clients felt the need to use a condom.

One of FSW also expressed a similar experience:

*'I spent last night with a client in a restaurant. I asked him to use a condom while having sex. He used one the first time but refused afterwards saying he had paid a huge amount for all night sex. He assured me he did not have HIV and was tested regularly' *(FSW ID 4, age 39)

Most FSWs said that it was the clients who decided irrespective of suggesting use a condom by the clients during their last sex. One FSW who had a sex with foreigners reported that her clients offered condoms while having sex with her.

She added thus:

*'My client (foreigner) wanted to use condom. Foreigners mostly ask to use condoms' *(FSW ID 7, age 42)

One FSW, who had obtained School Leaving Certificate (SLC) education, reported that she and her clients had jointly decided to use condoms. One younger and relatively educated FSW said confidently that she proposes her clients to use condom.

She stated in this way:

*'I always ask them (clients) to use condom, He did not refuse it' *(FSW ID 12, age 24)

One FSW whose husband was HIV positive never used condoms with her clients. She expressed her frustration towards life as a reason for not using condoms as follows:

*'My husband has been tested HIV positive. I can't say tell him to use a condom. I have checked for HIV but I am not positive yet. As anyone has to die one day......no one is immortal. So I don't press clients to use condom' *(FSW ID 1, age 28)

### Low self-efficacy and poverty

Powerlessness and poverty were frequently reported as the reasons behind the non-use of condoms by the FSWs. One FSW expressed her faithfulness of clients and her powerlessness in the following way:

*'Most of the clients say 'I love you, why do you not love me...? It is so difficult to ignore them. We are here for making money. After all, the clients pay us for sex, not to argue with us for using condoms. We have no other options...' *(FSW ID1, age 28)

One of the FSW described client's love and stated the following quote:

'He did not use condom last time because he did not like it. I reminded him but he said to me,' if we have a virus in us we will die together, why do you worry?'

(FSW ID 2, age 19)

Another FSW explained the risks she had to take in the light of the commercial relationship between the pimps and the clients.

*'The clients told me that he has no HIV. As he had paid for sex and I am also paid by my owner to provide services to the clients. I got this place here, after searching for a long time. If I lose this job I can't survive, and my family has to suffer' *(FSW ID 4, age 39)

Another FSW expressed her low power to negotiation for condoms use:

*'I have no power to force him (clients) to use condoms because I am here for this job. I have to keep my customers happy at all the time. We have to accept their demands; otherwise we need to leave our job' *(FSW ID 11, age 30)

Another FSW expressed the reason for not using condom with helpful clients:

*'I have a few regular clients who I have an attachment for. They love me and come to me with me they don't want sex using condoms. I never press them as I am a widow. They have helped me in many ways and in raising my kids*. So I cannot insist them using a condom' (FSW ID 7, age 42)

Different from others, a 30 year old literate FSW confidently said:

*'I use condoms with all my clients to protect me from STIs and HIV. One day when I asked a client to use a condom he refused at first. I denied having sex with him. Finally, he agreed to use condom. From then, I knew that if we become strong and can resist the clients, it is certain that they must agree' *(FSW ID 15, age 30)

### Condom use with paying clients/non-paying partners

The FSWs who we interviewed did not use condoms for various reasons: Clients' refusal due to their perception that a condom reduces pleasure during sex, some FSWs offering sex without a condom, FSW's misconception that well off and healthy clients and familiar men or boyfriends do not harbour STI and HIV were reported as the reasons for not using condoms.

*'They are big people, wealthy, have good education and good looking. They are superior to us. Some are our boyfriends and come to us regularly. For them we do not ask to use a condom. They also do not ask for us' *(FSW ID 8, age 30)

However, a FSW reported that most of the time clients make the decisions not to using a condom. One FSW stated her views in this way:

*'If we force them to use condom, they might refuse and turn to other FSWs who agree to sex without a condom. It will affect our job and earning. So it is up to them to decide whether or not to use condom' *(FSW ID 6, age 22)

One FSW expressed her feeling in this way:

*'He (client) did not like to use a condom. He flatly said ''I come here for entertainment'' If I do not get satisfaction, why should I come here and spend money? *'(FSW ID 13, age 22)

One or two FSWs initially claimed they used condoms every time. But later on they revealed that they really did not use a condom while they had sex with business men (rich people), artists, educated, apparently smart and healthy looking clients. They reported that they recognised the clients based on their personality, behaviours and the amount they pay. An FSW who had regular clients for a longer period was not using condoms at all. She expressed her view as follows:

*'I do not use condom with him (client) because he comes to me regularly. He is quite educated. He told me that he really loves me and wants to be fully relaxed with me. He pays me well' *(FSW ID 8, age 30)

### Condom use with non-paying partners

Of the 15 FSWs interviewed, three were single, five were living with boyfriends, and seven were living with husband. Almost all cohabitating FSWs reported that asking their regular partners to use condoms was culturally inappropriate and viewed as a denial to have sex.

They reported that they never asked their regular partners to use condoms except for family planning purposes. They had too low SE to force these non-paying partners to use condoms against their will. One FSW, who is a peer educator and living with an HIV-positive husband, reported that she did not use condoms with her clients, or with her husband, she said that she knew the risk but could not do anything to enforce condom use from following statement:

*'I know the danger of having sex without condoms. My husband is a HIV-positive... How can a wife ask her husband to use a condom? It is better to die than resist a husband... And how can I tell this (his HIV status) to my clients? I am helpless... As everyone has to die one day, and no one is immortal, I do not press my clients to use condoms' *(FSW ID 1, age 28)

Another FSW expressed her difficulties as:

*'If I tell my husband to use a condom he will not trust me, and he might leave me in trouble. I want to tell him (my husband) to use a condom but I cannot do this in our culture. We do not use condoms between husband and wife except for family planning purposes. If I ask he will guess my naughty work and leave me in chaos' *(FSW ID 10, age 26)

One FSW reported her fear of violence:

*'My husband is unemployed. He drinks a lot and often beats me up. He acts violently if I ask him to use condoms. I do not want to make it worse' *(FSW ID 6, age 22)

Another FSW stated her believe following way:

*'My boyfriend never uses a condom with me and I also accept this because in a true relationship there is no need to wear condoms. We tested our blood together last time. Both of us are HIV-negative. Except for family planning, it is not considered good to use condoms with a husband or close partner' *(FSW ID 11, age 30)

### Police harassment

Most of the FSWs claimed or perceived a threat that they could be arrested by the police and kept in jail if their identity as sex workers was discovered. A few FSWs reported that due to police raids for condoms (if they had condoms on their possession, they were 'charged' as sex workers), they no longer carry condoms with them. Most FSWs, when asked if they were carrying condoms on their possession, said no because they feared that the police would search their bags and find them. A typical answer to the question; "Do you keep condoms with you?" was:

*'No I don't.....if the police checks my bags searching for a bomb during war, it is sure he will take me to jail. For them both bomb and condoms are similar, both are illegal' *(FSW ID 9, age 25)

Even peer-educators, who have identity cards that should protect them from police harassment, are still harassed for example:

*'The police took me into custody for a couple of months after they found condoms with me. They beat me up with a stick. I had many bruises on my body. At one point I lost my capacity to stand. They also stole all my money from my purse' *(FSW ID 15, age 30)

One of FSW went on to complain that it was not only the police that harassed them, but also the Maoist rebels:

*'Nowadays some males who claim to be Maoist have also started torturing us. One of my friends was caught by them, beaten up in a public park ... She was made to undress there. As a result of this torture, she became critically ill. We FSWs are supporting her treatment now' *(FSW ID 15, age 30)

## Discussion

FSWs were relaxed and tended not to use condoms with apparently healthy, young, educated and wealthier clients. They rarely used condoms in personal sexual relationships with non-paying partners and perceived that foreigners could be safe to have unprotected sex. This, too, confirms UNAID's findings [26]. Almost all FSWs reported the reason behind non-use of condoms was a financial problem and low self-efficacy to convince clients, similar to the findings of Gysels & Nnalusiba [27] and [28-30]. This is similar to a study conducted in Nepal which found FSWs were reluctant to use condoms with physically healthy good looking and intimate clients [31].This finding is consistent with another study where those FSWs seemed reluctant to use condoms with a client in a close personal relationship [32].

Only three out of 15 FSWs in the in-depth interviews had carried condoms with them. This finding was confirmed during observation in cabin restaurants and massage centres. Despite claims by establishment owners, it was also confirmed through observation of the cabin and massage centres by the researcher. There were no condoms at these places as claimed by the owners.

FSW's use of condom was rooted in their concern for ensuring financial gain and earning their livelihoods. Sex workers strove to mitigate disruption and harassment by police, and to maintain their emotional and physical well-being. Attitudes to sexual health risks were shaped by these driving motivations, and thus can only be understood when seen through sex workers own world view. Within this, behaviours that are "risky" from a sexual health perspective such as accepting all clients, including those who refuse to wear condoms. Past studies in other countries show varying rates of condom use during a paid sexual transaction. For example, Wong *et al. *found that 80% of Cambodian FSWs used condoms consistently [33], when clients offered to pay more for sex without a condom, FSWs tended to accept the cash over the protection [34,35].

The perceived risk of HIV was found to be an important trigger among FSWs negotiating condom use with clients. Women were aware that proper condom use was an effective preventive measure for HIV [36]. Unfortunately, condoms were not always used with intimate non-paying partners or clients. Fear of clients and intimate partner's violence [37-39] and low sexual marketability resulted in client unwillingness to use condoms. Low socioeconomic status and subsequent lack of negotiation capacity or low SE, dependence on smoking, alcohol use, and misperceptions about client appearance in relation to their STI/HIV status, were identified as drivers for women to perform sex without condoms [40].

Additionally, lack of autonomy in a society that values decisions of men over the rights or well-being of women was an influencing factor. Confounding factors are the high mobility of FSWs, leading to increased health risks and difficulty conducting follow-ups, as well as the illegal status of sex work [41,42].

Finally, this qualitative study among the FSWs in Kathmandu Valley, Nepal found a clear hierarchy of concerns within the community. Sex workers took pride in their ability to contribute to daily hand to mouth concerns and their families' incomes and viewed their role as earners as of foremost importance. This finding is supported by a study carried out in Vietnamese sex workers in Cambodia and Indonesia [43,44]. Threats to the potential to fulfil these obligations proved to be among sex workers' most serious preoccupations and led them to adopt various strategies including maximizing numbers of clients and choosing clients who paid well but often refused to use condoms. High levels of violence faced in the society from all kinds of authority, even facing intimate partners and police violence instead of security [45,46]. Fear of police harassment, violence, instability and disruption to normal work conditions were frequently reported. This finding was confirmed by Meyhew et al. in a study among female sex workers in Rawalpindi Pakistan [39].

## Study Limitations

The findings presented in this paper are based on self-reporting by the FSWs from within the Kathmandu Valley. As sex work is illegal in Nepal, FSWs may have provided some inaccurate or incomplete information because there were difficulties in identifying a sampling frame of FSWs and secure venues where interviews could be conducted due to the fear of media exposure and previous episodes of confidentiality violation. FSWs are also highly mobile due to the fear of police and other armed personnel.

## Conclusion

This study considers that sex workers were highly vulnerable due to low Self-Efficacy (SE) for their health compromising behaviours, anxiety, fear of life threatened disease like HIV, poor physical, mental and social health, lower levels of education and livelihood problems. Poverty, gender inequality, lack of empowerment [45,46] and low social status diminishes an individual's ability to act on positive intentions to use condoms with clients. Complex problems and illegal status of sex work hampers interventions targeting the FSWs. Economic empowerment of women and other structural interventions may provide a more sustainable means of STI and HIV prevention by strengthening the ability of communities to help individuals to reduce these risks and vulnerabilities [47-49]. Interventions that combine community solidarity and government action have proven effective in reducing risk among FSWs in other settings [50]. It is therefore suggested that implementation of FSW empowerment and behaviour, targeted education, information and communication programmes along with improved provision of health services for the FSWs would reinforce the relevant policy changes will also uplift the SE of FSWs [51], P.217].

## Abbreviations

(SACTS): STI/AIDS Counselling and Training Service is a non-profit non-governmental organisation; (FSWs): Female sex workers, (FHI): Family Health International; (NCASC): National Centre for AIDS and STD Control; (STI): sexually transmitted infections, (SLC): School Leaving Certificate, (SE): Self-efficacy.

## Competing interests

The authors declare that they have no competing interests.

## Authors' contributions

LG participated in the design, carried out the whole study, interviewed the participant, prepared the dataset, analysed and prepared all the results and drafted the manuscript. WCSS redrafted the manuscript and contributed to design of the analysis. ERvT supervised the data analysis, design of the study and redrafted the manuscript and made important final modifications. RD organised to the interviews in confidential local setting and redrafted the manuscript. NPL facilitated interviews, organising a data set, and helped to redraft the manuscript. All authors read and approved the final manuscript.

## Pre-publication history

The pre-publication history for this paper can be accessed here:

http://www.biomedcentral.com/1472-6874/11/42/prepub
